# On a more accurate Hardy-Mulholland-type inequality

**DOI:** 10.1186/s13660-017-1442-8

**Published:** 2017-07-12

**Authors:** Bicheng Yang, Qiang Chen

**Affiliations:** 1 Department of Mathematics, Guangdong University of Education, Guangzhou, Guangdong 510303 P.R. China; 2 Department of Computer Science, Guangdong University of Education, Guangzhou, Guangdong 510303 P.R. China

**Keywords:** 26D15, 47A07, Mulholland-type inequality, weight coefficient, equivalent form, reverse, operator

## Abstract

By using the way of weight coefficients, the technique of real analysis, and Hermite-Hadamard’s inequality, a more accurate Hardy-Mulholland-type inequality with multi-parameters and a best possible constant factor is given. The equivalent forms, the reverses, the operator expressions and some particular cases are considered.

## Introduction

Assuming that $p > 1$, $\frac{1}{p} + \frac{1}{q} = 1$, $a_{m},b_{n} \ge 0$, $a = \{ a_{m}\}_{m = 1}^{\infty} \in l^{p}$, $b = \{ b_{n}\}_{n = 1}^{\infty} \in l^{q}$, $\Vert a \Vert _{p} = (\sum_{m = 1}^{\infty} a_{m}^{p} )^{\frac{1}{p}} > 0$, and $\Vert b \Vert _{q} > 0$, we have the following Hardy-Hilbert’s inequality with the best possible constant $\frac{\pi}{\sin (\pi /p)}$ (cf. [1], Theorem 315):

1 $$ \sum_{m = 1}^{\infty} \sum _{n = 1}^{\infty} \frac{a_{m}b{}_{n}}{m + n} < \frac{\pi}{\sin (\pi /p)} \Vert a \Vert _{p} \Vert b \Vert _{q}. $$

A more accurate inequality of (1) is given as follows (cf. [1], Th. 323 and [2]):

2 $$ \sum_{m = 1}^{\infty} \sum _{n = 1}^{\infty} \frac{a_{m}b{}_{n}}{m + n - \alpha} < \frac{\pi}{\sin (\pi /p)} \Vert a \Vert _{p} \Vert b \Vert _{q}\quad (0 \le \alpha \le 1), $$

where the constant factor $\frac{\pi}{\sin (\pi /p)}$ is still the best possible.

Also we have the following Mulholland’s inequality similar to (1) with the same best possible constant factor $\frac{\pi}{\sin (\pi /p)}$ (cf. [3] or [1], Th. 343, replacing $\frac{a_{m}}{m}$, $\frac{b_{n}}{n}$ by $a_{m}$, $b_{n}$):

3 $$ \sum_{m = 2}^{\infty} \sum _{n = 2}^{\infty} \frac{a_{m}b{}_{n}}{\ln mn} < \frac{\pi}{\sin (\pi /p)} \Biggl( \sum_{m = 2}^{\infty} \frac{a_{m}^{p}}{m^{1 - p}} \Biggr)^{\frac{1}{p}} \Biggl( \sum_{n = 2}^{\infty} \frac{b_{n}^{q}}{n^{1 - q}} \Biggr)^{\frac{1}{q}}. $$

Inequalities (1)-(3) are important in analysis and its applications (cf. [1, 2, 4–18]).

Suppose that $\mu_{i},\upsilon_{j} > 0$ ($i,j \in \mathbb{N} = \{ 1,2, \ldots \}$),

4 $$ U_{m} = \sum_{i = 1}^{m} \mu_{i},\qquad V_{n} = \sum_{j = 1}^{n} \upsilon_{j}\quad (m,n \in \mathbb{N}), $$

we have the following Hardy-Hilbert-type inequality (cf. [1], Theorem 321, replacing $\mu_{m}^{1/q}a_{m}$ and $\upsilon_{n}^{1/p}b_{n}$ by $a_{m}$ and $b_{n}$): If $a_{m},b_{n} \ge 0$, $0 < \sum_{m = 1}^{\infty} \frac{a_{m}^{p}}{m^{p - 1}} < \infty$, $0 < \sum_{n = 1}^{\infty} \frac{b_{n}^{q}}{n^{q - 1}} < \infty$, then

5 $$ \sum_{m = 1}^{\infty} \sum _{n = 1}^{\infty} \frac{a_{m}b{}_{n}}{U_{m} + V_{n}} < \frac{\pi}{\sin (\pi /p)} \Biggl( \sum_{m = 1}^{\infty} \frac{a_{m}^{p}}{\mu_{m}^{p - 1}} \Biggr)^{\frac{1}{p}} \Biggl( \sum_{n = 1}^{\infty} \frac{b_{n}^{q}}{\upsilon_{n}^{q - 1}} \Biggr)^{\frac{1}{q}}. $$

For $\mu_{i} = \upsilon_{j} = 1$ ($i,j \in \mathbb{N}$), inequality (5) reduces to (1).

In 2015, Yang [19] gave an extension of (5) as follows: If $0 < \lambda_{1},\lambda_{2} \le 1$, $\lambda_{1} + \lambda_{2} = \lambda$, $\{ \mu_{m}\}_{m = 1}^{\infty}$ and $\{ \upsilon_{n}\}_{n = 1}^{\infty}$ are positive and decreasing, with $U_{\infty} = V_{\infty} = \infty$, then we have the following inequality with the best possible constant factor $\pi /\sin (\frac{\pi \lambda_{1}}{\lambda} )$:

6 $$ \sum_{m = 1}^{\infty} \sum _{n = 1}^{\infty} \frac{a_{m}b{}_{n}}{U_{m}^{\lambda} + V_{n}^{\lambda}} < \frac{\pi}{ \lambda \sin (\frac{\pi \lambda_{1}}{\lambda} )} \Biggl[ \sum_{m = 1}^{\infty} \frac{U_{m}^{p(1 - \lambda_{1}) - 1}a_{m}^{p}}{\mu_{m}^{p - 1}} \Biggr]^{\frac{1}{p}} \Biggl[ \sum_{n = 1}^{\infty} \frac{V_{n}^{q(1 - \lambda_{2}) - 1}b_{n}^{q}}{\upsilon_{n}^{q - 1}} \Biggr]^{\frac{1}{q}}. $$

In this paper, by using the way of weight coefficients, the technique of real analysis, and Hermite-Hadamard’s inequality, a new Hardy-Mulholland-type inequality with a best possible constant factor is given as follows: If $\mu_{1} = \upsilon_{1} = 1$, $\{ \mu_{m}\}_{m = 1}^{\infty}$ and $\{ \upsilon_{n}\}_{n = 1}^{\infty}$ are positive and decreasing, with $U_{\infty} = V_{\infty} = \infty$, we have the following inequality:

7 $$ \sum_{m = 2}^{\infty} \sum _{n = 2}^{\infty} \frac{a_{m}b{}_{n}}{\ln U_{m}V_{n}} < \frac{\pi}{\sin (\frac{\pi}{p})} \Biggl[ \sum_{m = 2}^{\infty} \biggl( \frac{U_{m}}{\mu_{m + 1}}\biggr)^{p - 1}a_{m}^{p} \Biggr]^{\frac{1}{p}} \Biggl[ \sum_{n = 2}^{\infty} \biggl(\frac{V_{n}}{\upsilon_{n + 1}}\biggr)^{q - 1}b_{n}^{q} \Biggr]^{\frac{1}{q}}, $$

which is an extension of (3). Moreover, the more accurate inequality of (7) and its extension with multi-parameters and the best possible constant factors are obtained. The equivalent forms, the reverses, the operator expressions and some particular cases are considered.

## Some lemmas and an example

In the following, we agree that $p \ne 0,1$, $\frac{1}{p} + \frac{1}{q} = 1$, $- 1 < \gamma \le 0$, $0 < \lambda_{1},\lambda_{2} < 1$, $\lambda_{1} + \lambda_{2} = \lambda$, $\mu_{i},\upsilon_{j} > 0$ ($i,j \in \mathbb{N}$), with $\mu_{1} = \upsilon_{1} = 1$, $U_{m}$ and $V_{n}$ are defined by (4),

$$\frac{1}{1 + \frac{\mu_{2}}{2}} \le \alpha \le 1,\qquad \frac{1}{1 + \frac{\upsilon_{2}}{2}} \le \beta \le 1, $$

$a_{m},b_{n} \ge 0$, $\Vert a \Vert _{p,\Phi_{\lambda}}: = (\sum_{m = 2}^{\infty} \Phi_{\lambda} (m)a_{m}^{p})^{\frac{1}{p}}$ and $\Vert b \Vert _{q,\Psi_{\lambda}}: = (\sum_{n = 2}^{\infty} \Psi_{\lambda} (n)b_{n}^{q})^{\frac{1}{q}}$, where

8 $$ \begin{gathered} \Phi_{\lambda} (m): = \biggl(\frac{U_{m}}{\mu_{m + 1}}\biggr)^{p - 1}(\ln \alpha U_{m})^{p(1 - \lambda_{1}) - 1}, \\ \Psi_{\lambda} (n): = \biggl(\frac{V_{n}}{\upsilon_{n + 1}}\biggr)^{q - 1}(\ln \beta V_{n})^{q(1 - \lambda_{2}) - 1}\quad \bigl(m,n \in \mathbb{N}\backslash \{ 1\} \bigr). \end{gathered} $$

### Lemma 1

*If* $n \in \mathbb{N}\backslash \{ 1\}$, $a \in (n - \frac{1}{2},n)$, $f(x)$ *is continuous in* $(n - \frac{1}{2}, n + \frac{1}{2})$, *and* $f'(x)$ *is strictly increasing in the intervals* $(n - \frac{1}{2},a)$, $(a,n)$ *and* $(n,n + \frac{1}{2})$, *respectively*, *satisfying*

9 $$ f'(a - 0) \le f'(a + 0),\qquad f'(n - 0) \le f'(n + 0), $$

*then we have the following Hermite*-*Hadamard’s inequality* (*cf*. [20]).

### Proof

In view of $f'(n - 0) \le f'(n + 0) = \lim_{x \to n^{ +}} f'(x)$ is finite, we set the linear function $g(x)$ as follows:

$$g(x): = f'(n - 0) (x - n) + f(n),\quad x \in \biggl[n - \frac{1}{2}, n + \frac{1}{2}\biggr]. $$

Since $f'(x)$ is strictly increasing in $[n - \frac{1}{2},a)$ and $(a,n)$, then for $x \in [n - \frac{1}{2},a)$,

$$f'(x) < \lim_{x \to a^{ -}} f'(x) = f'(a - 0) \le f'(a + 0) < f'(n - 0); $$

for $x \in (a,n)$, $f'(x) < \lim_{x \to n^{ -}} f'(x) = f'(n - 0)$. Hence,

$$\bigl(f(x) - g(x)\bigr)' = f'(x) - f'(n - 0) < 0,\quad x \in \biggl(n - \frac{1}{2},a\biggr) \cup (a,n). $$

Since $f(x)-g(x)$ is continuous in $(n - \frac{1}{2},n]$ with $f(n)-g(n)=0$, it follows that

$$f(x) - g(x) > 0,\quad x \in \biggl(n - \frac{1}{2},n\biggr). $$

In the same way, since $f'(x)$ is strictly increasing in $(n,n + \frac{1}{2})$, then for $x \in (n,n + \frac{1}{2})$, $f'(x) > f'(n + 0) \ge f'(n - 0)$. Hence,

$$\bigl(f(x) - g(x)\bigr)' = f'(x) - f'(n - 0) > 0,\quad x \in \biggl(n,n + \frac{1}{2}\biggr). $$

Since $f(x)-g(x)$ is continuous in $[n,n + \frac{1}{2})$ with $f(n)-g(n)=0$, it follows that

$$f(x) - g(x) > 0,\quad x \in \biggl(n,n + \frac{1}{2}\biggr). $$

Therefore, we have $f(x) - g(x) > 0$, $x \in (n - \frac{1}{2},n + \frac{1}{2})\backslash \{ n\}$. Then we find

$$\int_{n - \frac{1}{2}}^{n + \frac{1}{2}} f(x)\,dx > \int_{n - \frac{1}{2}}^{n + \frac{1}{2}} g(x)\,dx = f(n), $$

namely, (9) follows. The lemma is proved. □

### Note

With the assumptions of Lemma 1, if (i) $a \in (n,n + \frac{1}{2})$, $f'(x)$ is strictly increasing in the intervals $(n - \frac{1}{2},n)$, $(n,a)$ and $(a,n + \frac{1}{2})$, respectively, or (ii) $a = n$, $f'(x)$ is strictly increasing in the intervals $(n - \frac{1}{2},n)$ and $(n,n + \frac{1}{2})$, respectively, then in the same way, we still can obtain (9).

### Example 1

$\{ \mu_{m}\}_{m = 1}^{\infty}$ and $\{ \upsilon_{n}\}_{n = 1}^{\infty}$ are decreasing, we set functions $\mu (t): = \mu_{m}$, $t \in (m - 1,m]$ ($m \in \mathbb{N}$), $\upsilon (t): = \upsilon_{n}$, $t \in (n - 1,n]$ ($n \in \mathbb{N}$), and

10 $$ U(x): = \int_{0}^{x} \mu (t)\,dt\quad (x \ge 0),\qquad V(y): = \int_{0}^{y} \upsilon (t)\,dt\quad (y \ge 0). $$

Then it follows that $U(m) = U_{m}$, $V(n) = V_{n}$, $U(\infty ) = U_{\infty}$, $V(\infty ) = V_{\infty}$ and

$$\begin{gathered} U'(x) = \mu (x) = \mu_{m},\quad x \in (m - 1,m), \\ V'(y) = \upsilon (y) = \upsilon_{n},\quad y \in (n - 1,n)\ (x,y \in \mathbb{N}). \end{gathered} $$

For $0 < \lambda \le 1$, $- 1 < \gamma \le 0$, we set

11 $$ k_{\lambda} (x,y): = \frac{1}{x^{\lambda} + y^{\lambda} + \gamma \vert x^{\lambda} - y^{\lambda} \vert }\quad (x,y > 0). $$

We find

12 $$ \begin{aligned}[b] 0 &< K_{\gamma} (\lambda_{1}): = \int_{0}^{\infty} k_{\lambda} (1,t)t^{\lambda_{2} - 1} \,dt = \int_{0}^{\infty} k_{\lambda} (t,1)t^{\lambda_{1} - 1} \,dt \\ &= \int_{0}^{\infty} \frac{t^{\lambda_{1} - 1}}{t^{\lambda} + 1 + \gamma \vert t^{\lambda} - 1 \vert } \,dt = \int_{0}^{1} \frac{t^{\lambda_{1} - 1} + t^{\lambda_{2} - 1}}{1 + \gamma + (1 - \gamma )t^{\lambda}} \,dt \\ &\le \int_{0}^{1} \frac{t^{\lambda_{1} - 1} + t^{\lambda_{2} - 1}}{1 + \gamma} \,dt = \frac{1}{1 + \gamma} \biggl(\frac{1}{\lambda_{1}} + \frac{1}{\lambda_{2}}\biggr) < \infty, \end{aligned} $$

namely, $K_{\gamma} (\lambda_{1}) \in \mathbb{R}_{ +}$. In the following, we express $K_{\gamma} (\lambda_{1})$ in other forms.

(i) For $\gamma = 0$, we obtain

13 $$ K_{0}(\lambda_{1}) = \int_{0}^{\infty} \frac{t^{\lambda_{1} - 1}}{t^{\lambda} + 1} \,dt = \frac{1}{\lambda} \int_{0}^{\infty} \frac{v^{(\lambda_{1}/\lambda ) - 1}}{v + 1} \,dv = \frac{\pi}{\lambda \sin (\frac{\pi \lambda_{1}}{\lambda} )}; $$

(ii) for $- 1 < \gamma < 0$, $0 < \frac{1 + \gamma}{1 - \gamma} < 1$, by the Lebesgue term by term integration theorem (cf. [21]), we find

14 $$\begin{aligned} K_{\gamma} (\lambda_{1}) &= \frac{1}{1 - \gamma} \int_{0}^{1} \frac{t^{ - \lambda_{2} - 1} + t^{ - \lambda_{1} - 1}}{\frac{1 + \gamma}{1 - \gamma} t^{ - \lambda} + 1} \,dt \\ &\mathop{ =} ^{v = \frac{1 + \gamma}{1 - \gamma} t^{ - \lambda}} \frac{1}{\lambda (1 - \gamma )} \int_{\frac{1 + \gamma}{1 - \gamma}}^{\infty} \frac{1}{v + 1} \biggl[ \biggl( \frac{1 + \gamma}{1 - \gamma} \biggr)^{\frac{\lambda_{1}}{\lambda} - 1}v^{\frac{\lambda_{2}}{\lambda} - 1} + \biggl( \frac{1 + \gamma}{1 - \gamma} \biggr)^{\frac{\lambda_{2}}{\lambda} - 1}v^{\frac{\lambda_{1}}{\lambda} - 1} \biggr] \,dv \\ &= \frac{1}{\lambda (1 - \gamma )}\biggl(\frac{1 + \gamma}{1 - \gamma} \biggr)^{\frac{\lambda_{1}}{\lambda} - 1} \int_{0}^{\infty} \frac{v^{\frac{\lambda_{2}}{\lambda} - 1}}{v + 1} \,dv + \frac{1}{\lambda (1 - \gamma )}\biggl(\frac{1 + \gamma}{1 - \gamma} \biggr)^{\frac{\lambda_{2}}{\lambda} - 1} \int_{0}^{\infty} \frac{v^{\frac{\lambda_{1}}{\lambda} - 1}}{v + 1} \,dv \\ &\quad {}- \frac{1}{\lambda (1 - \gamma )} \int_{0}^{\frac{1 + \gamma}{1 - \gamma}} \frac{1}{v + 1} \biggl[ \biggl( \frac{1 + \gamma}{1 - \gamma} \biggr)^{\frac{\lambda_{1}}{\lambda} - 1}v^{\frac{\lambda_{2}}{\lambda} - 1} + \biggl( \frac{1 + \gamma}{1 - \gamma} \biggr)^{\frac{\lambda_{2}}{\lambda} - 1}v^{\frac{\lambda_{1}}{\lambda} - 1} \biggr]\,dv \\ & = \frac{1}{\lambda (1 - \gamma )}\biggl(\frac{1 + \gamma}{1 - \gamma} \biggr)^{\frac{\lambda_{1}}{\lambda} - 1} \frac{\pi}{\sin (\frac{\pi \lambda_{2}}{\lambda} )} + \frac{1}{\lambda (1 - \gamma )}\biggl(\frac{1 + \gamma}{1 - \gamma} \biggr)^{\frac{\lambda_{2}}{\lambda} - 1}\frac{\pi}{\sin (\frac{\pi \lambda_{1}}{\lambda} )} \\ &\quad {}- \frac{1}{\lambda (1 - \gamma )} \int_{0}^{\frac{1 + \gamma}{1 - \gamma}} \sum_{k = 0}^{\infty} ( - 1)^{k}v^{k} \biggl[ \biggl(\frac{1 + \gamma}{1 - \gamma} \biggr)^{\frac{\lambda_{1}}{\lambda} - 1}v^{\frac{\lambda_{2}}{\lambda} - 1} + \biggl(\frac{1 + \gamma}{1 - \gamma} \biggr)^{\frac{\lambda_{2}}{\lambda} - 1}v^{\frac{\lambda_{1}}{\lambda} - 1} \biggr]\,dv \\ &= \frac{1}{\lambda (1 - \gamma )} \biggl[ \biggl(\frac{1 + \gamma}{1 - \gamma} \biggr)^{\frac{\lambda_{1}}{\lambda} - 1} + \biggl(\frac{1 + \gamma}{1 - \gamma} \biggr)^{\frac{\lambda_{2}}{\lambda} - 1} \biggr]\frac{\pi}{\sin (\frac{\pi \lambda_{1}}{\lambda} )} \\ &\quad {}- \frac{1}{\lambda (1 - \gamma )} \int_{0}^{\frac{1 + \gamma}{1 - \gamma}} \sum_{k = 0}^{\infty} \bigl(v^{2k} - v^{2k + 1}\bigr) \biggl[ \biggl(\frac{1 + \gamma}{1 - \gamma} \biggr)^{\frac{\lambda_{1}}{\lambda} - 1}v^{\frac{\lambda_{2}}{\lambda} - 1} \\ &\quad {} + \biggl(\frac{1 + \gamma}{1 - \gamma} \biggr)^{\frac{\lambda_{2}}{\lambda} - 1}v^{\frac{\lambda_{1}}{\lambda} - 1} \biggr]\,dv \\ & = \frac{1}{1 + \gamma} \biggl[ \biggl(\frac{1 + \gamma}{1 - \gamma} \biggr)^{\frac{\lambda_{1}}{\lambda}} + \biggl(\frac{1 + \gamma}{1 - \gamma} \biggr)^{\frac{\lambda_{2}}{\lambda}} \biggr]\frac{\pi}{\lambda \sin (\frac{\pi \lambda_{1}}{\lambda} )} \\ &\quad {}- \frac{1}{\lambda (1 + \gamma )} \int_{0}^{\frac{1 + \gamma}{1 - \gamma}} \sum_{k = 0}^{\infty} \bigl(v^{2k} - v^{2k + 1}\bigr) \biggl[ \biggl(\frac{1 + \gamma}{1 - \gamma} \biggr)^{\frac{\lambda_{1}}{\lambda}} v^{\frac{\lambda_{2}}{\lambda} - 1} + \biggl(\frac{1 + \gamma}{1 - \gamma} \biggr)^{\frac{\lambda_{2}}{\lambda}} v^{\frac{\lambda_{1}}{\lambda} - 1} \biggr]\,dv \\ &= \frac{1}{1 + \gamma} \biggl[ \biggl(\frac{1 + \gamma}{1 - \gamma} \biggr)^{\frac{\lambda_{1}}{\lambda}} + \biggl(\frac{1 + \gamma}{1 - \gamma} \biggr)^{\frac{\lambda_{2}}{\lambda}} \biggr]\frac{\pi}{\lambda \sin (\frac{\pi \lambda_{1}}{\lambda} )} \\ &\quad {}- \frac{1}{\lambda (1 + \gamma )}\sum_{k = 0}^{\infty} \int_{0}^{\frac{1 + \gamma}{1 - \gamma}} ( - 1)^{k} v^{k} \biggl[ \biggl(\frac{1 + \gamma}{1 - \gamma} \biggr)^{\frac{\lambda_{1}}{\lambda}} v^{\frac{\lambda_{2}}{\lambda} - 1} + \biggl(\frac{1 + \gamma}{1 - \gamma} \biggr)^{\frac{\lambda_{2}}{\lambda} - 1}v^{\frac{\lambda_{1}}{\lambda} - 1} \biggr]\,dv \\ &= \frac{1}{1 + \gamma} \biggl[ \biggl(\frac{1 + \gamma}{1 - \gamma} \biggr)^{\frac{\lambda_{1}}{\lambda}} + \biggl(\frac{1 + \gamma}{1 - \gamma} \biggr)^{\frac{\lambda_{2}}{\lambda}} \biggr]\frac{\pi}{\lambda \sin (\frac{\pi \lambda_{1}}{\lambda} )} \\ &\quad {}- \frac{1}{1 + \gamma} \sum_{k = 0}^{\infty} ( - 1)^{k}\biggl(\frac{1 + \gamma}{ 1 - \gamma} \biggr)^{k + 1} \biggl( \frac{1}{\lambda k + \lambda_{2}} + \frac{1}{\lambda k + \lambda_{1}} \biggr); \end{aligned}$$

(iii) for $\lambda_{1} = \lambda_{2} = \frac{\lambda}{2}$, $- 1 < \gamma < 0$, we find

15 $$ \begin{aligned}[b] K_{\gamma} \biggl(\frac{\lambda}{2} \biggr) &= 2 \int_{0}^{1} \frac{t^{(\lambda /2) - 1}}{1 + \gamma + (1 - \gamma )t^{\lambda}} \,dt\mathop{ =} ^{u = (\frac{1 - \gamma}{1 + \gamma} t^{\lambda} )^{\frac{1}{2}}} \frac{4}{\lambda (1 + \gamma )}\biggl(\frac{1 + \gamma}{1 - \gamma} \biggr)^{\frac{1}{2}} \int_{0}^{(\frac{1 - \gamma}{1 + \gamma} )^{\frac{1}{2}}} \frac{du}{1 + u^{2}} \\ &= \frac{4}{\lambda (1 + \gamma )}\biggl(\frac{1 + \gamma}{1 - \gamma} \biggr)^{\frac{1}{2}}\arctan \biggl(\frac{1 - \gamma}{1 + \gamma} \biggr)^{\frac{1}{2}}. \end{aligned} $$

For fixed $m \in \mathbb{N}\backslash \{ 1\}$, we define the function $f(y)$ as follows:

$$f(y): = k_{\lambda} \bigl(\ln \alpha U_{m},\ln \beta V(y)\bigr),\quad y \in \biggl(n - \frac{1}{2},n + \frac{1}{2}\biggr)\ \bigl(n \in \mathbb{N}\backslash \{ 1\} \bigr). $$

Then $f(y)$ is continuous in $(n - \frac{1}{2},n + \frac{1}{2})$ ($n \in \mathbb{N}\backslash \{ 1\} $). There exists a unified number $y_{0} > \frac{3}{2}$ satisfying $V(y_{0}) = \frac{\alpha}{\beta} U_{m}$.

(i) If $y_{0} \in (n - \frac{1}{2},n + \frac{1}{2})$, we find

$$f(y) = \textstyle\begin{cases} \frac{1}{(1 + \gamma )\ln^{\lambda} \alpha U_{m} + (1 - \gamma )\ln^{\lambda} \beta V(y)},&n - \frac{1}{2} < y < y_{0}, \\ \frac{1}{(1 - \gamma )\ln^{\lambda} \alpha U_{m} + (1 + \gamma )\ln^{\lambda} \beta V(y)},&y_{0} < y < n + \frac{1}{2}. \end{cases} $$

For $y_{0} \ne n$, we obtain for $y \ne n$ that

$$f'(y) = \textstyle\begin{cases} \frac{ - \lambda (1 - \gamma )V'(y)\ln^{\lambda - 1}\beta V(y)}{V(y)[(1 + \gamma )\ln^{\lambda} \alpha U_{m} + (1 - \gamma )\ln^{\lambda} \beta V(y)]^{2}},&n - \frac{1}{2} < y < y_{0}, \\ \frac{ - \lambda (1 + \gamma )V'(y)\ln^{\lambda - 1}\beta V(y)}{V(y)[(1 - \gamma )\ln^{\lambda} \alpha U_{m} + (1 + \gamma )\ln^{\lambda} \beta V(y)]^{2}},&y_{0} < y < n + \frac{1}{2}; \end{cases} $$

for $y_{0} \ne n$, we obtain for $y = n$ that

$$\begin{gathered} f'(n - 0) = \textstyle\begin{cases} \frac{ - \lambda (1 - \gamma )\upsilon_{n}\ln^{\lambda - 1}\beta V_{n}}{V_{n}[(1 + \gamma )\ln^{\lambda} \alpha U_{m} + (1 - \gamma )\ln^{\lambda} \beta V_{n}]^{2}},&n - \frac{1}{2} < y < y_{0}, \\ \frac{ - \lambda (1 + \gamma )\upsilon_{n}\ln^{\lambda - 1}\beta V_{n}}{V_{n}[(1 - \gamma )\ln^{\lambda} \alpha U_{m} + (1 + \gamma )\ln^{\lambda} \beta V_{n}]^{2}},&y_{0} < y < n + \frac{1}{2}, \end{cases}\displaystyle \\ f'(n + 0) = \textstyle\begin{cases} \frac{ - \lambda (1 - \gamma )\upsilon_{n + 1}\ln^{\lambda - 1}\beta V_{n}}{V_{n}[(1 + \gamma )\ln^{\lambda} \alpha U_{m} + (1 - \gamma )\ln^{\lambda} \beta V_{n}]^{2}},&n - \frac{1}{2} < y < y_{0}, \\ \frac{ - \lambda (1 + \gamma )\upsilon_{n + 1}\ln^{\lambda - 1}\beta V_{n}}{V_{n}[(1 - \gamma )\ln^{\lambda} \alpha U_{m} + (1 + \gamma )\ln^{\lambda} \beta V_{n}]^{2}},&y_{0} < y < n + \frac{1}{2}. \end{cases}\displaystyle \end{gathered} $$

Since $0 < \lambda \le 1$, $- 1 < \gamma \le 0$, $(1 - \gamma )\upsilon_{n} \ge (1 + \gamma )\upsilon_{n + 1}$, in view of the above results, we find $f'(n - 0) \le f'(n + 0)$ ($n \ne y_{0}$), and $f'(y)$ (<0) is strictly increasing in $(n - \frac{1}{2},y_{0})$, $(y_{0},n)$ and $(n,n + \frac{1}{2})$ for $y_{0} < n$ or in $(n - \frac{1}{2},n)$, $(n,y_{0})$ and $(y_{0}, n + \frac{1}{2})$ for $y_{0} > n$.

We obtain

$$\begin{aligned}& \begin{aligned} f'(y_{0} - 0) &= \frac{ - \lambda (1 - \gamma )V'(y_{0} - 0)\ln^{\lambda - 1}\beta V(y_{0})}{V(y_{0})[(1 + \gamma )\ln^{\lambda} \alpha U_{m} + (1 - \gamma )\ln^{\lambda} \beta V(y_{0})]^{2}}\\ & = \frac{ - \lambda (1 - \gamma )V'(y_{0} - 0)\ln^{\lambda - 1}\beta V(y_{0})}{V(y_{0})(2\ln^{\lambda} \alpha U_{m})^{2}}, \end{aligned} \\& \begin{aligned} f'(y_{0} + 0) &= \frac{ - \lambda (1 + \gamma )V'(y_{0} + 0)\ln^{\lambda - 1}\beta V(y_{0})}{V(y_{0})[(1 - \gamma )\ln^{\lambda} \alpha U_{m} + (1 + \gamma )\ln^{\lambda} \beta V(y_{0})]^{2}}\\ & = \frac{ - \lambda (1 + \gamma )V'(y_{0} + 0)\ln^{\lambda - 1}\beta V(y_{0})}{V(y_{0})(2\ln^{\lambda} \alpha U_{m})^{2}}. \end{aligned} \end{aligned}$$

Since for $y_{0} = n$, $V'(y_{0} - 0) = \upsilon_{n}$, $V'(y_{0} + 0) = \upsilon_{n + 1}$ and for $y_{0} \ne n$, $V'(y_{0} - 0) = V'(y_{0})$, then we have $\lambda (1 - \gamma )V'(y_{0} - 0) \ge \lambda (1 + \gamma )V'(y_{0} + 0)$, namely, $f'(y_{0} - 0) \le f'(y_{0} + 0)$.

(ii) If $y_{0} \notin (n - \frac{1}{2},n + \frac{1}{2})$, then it follows that $f'(y) = \frac{V'(y)}{V(y)}\frac{d}{dy}k_{\lambda} (\ln \alpha U_{m},\ln \beta V(y)) < 0$, $y \in (n - \frac{1}{2},n + \frac{1}{2})\backslash \{ n\}$. We still can find that

$$\begin{aligned} \frac{\upsilon_{n}}{V_{n}}\frac{d}{dy}k_{\lambda} \bigl(\ln \alpha U_{m},\ln \beta V(y)\bigr)\bigg\vert _{y = n} &= f'(n - 0) \\ &\le f'(n + 0) = \frac{\upsilon_{n + 1}}{V_{n}}\frac{d}{dy}k_{\lambda} \bigl(\ln \alpha U_{m},\ln \beta V(y)\bigr)\bigg\vert _{y = n}, \end{aligned} $$

and $f'(y)$ (<0) is strictly increasing in $(n - \frac{1}{2},n)$ and $(n,n + \frac{1}{2})$.

Therefore, $f(y)$ satisfies the conditions of Lemma 1 with Note. So does $g(y) = \frac{f(y)}{V(y)\ln^{1 - \lambda_{2}}\beta V(y)}$. Hence, by (9), we have

16 $$ \frac{k_{\lambda} (\ln \alpha U_{m},\ln \beta V_{n})}{V_{n}\ln^{1 - \lambda_{2}}\beta V_{n}} < \int_{n - \frac{1}{2}}^{n + \frac{1}{2}} \frac{k_{\lambda} (\ln \alpha U_{m},\ln \beta V(y))}{V(y)\ln^{1 - \lambda_{2}}\beta V(y)} \,dy\quad \bigl(n \in \mathbb{N}\backslash \{ 1\} \bigr). $$

### Definition 1

Define the following weight coefficients:

17 $$ \begin{gathered} \omega (\lambda_{2},m): = \sum_{n = 2}^{\infty} k_{\lambda} (\ln \alpha U_{m},\ln \beta V_{n}) \frac{\upsilon_{n + 1}\ln^{\lambda_{1}}\alpha U_{m}}{V_{n}\ln^{1 - \lambda_{2}}\beta V_{n}},\quad m \in \mathbb{N} \backslash \{ 1\}, \\ \varpi (\lambda_{1},n): = \sum_{m = 2}^{\infty} k_{\lambda} (\ln \alpha U_{m},\ln \beta V_{n}) \frac{\mu_{m + 1}\ln^{\lambda_{2}}\beta V_{n}}{U_{m}\ln^{1 - \lambda_{1}}\alpha U_{m}},\quad n \in \mathbb{N}\backslash \{ 1\}. \end{gathered} $$

### Lemma 2

*If* $\{ \mu_{m}\}_{m = 1}^{\infty}$ *and* $\{ \upsilon_{n}\}_{n = 1}^{\infty}$ *are decreasing and* $U_{\infty} = V_{\infty} = \infty$, *then for* $m,n \in \mathbb{N}\backslash \{ 1\}$, *we have the following inequalities*:

18 $$\begin{aligned}& \omega (\lambda_{2},m) < K_{\gamma} (\lambda_{1}), \end{aligned}$$

19 $$\begin{aligned}& \varpi (\lambda_{1},n) < K_{\gamma} (\lambda_{1}), \end{aligned}$$

*where* $K_{\gamma} (\lambda_{1})$ *is determined by* (12).

### Proof

For $y \in (n - \frac{1}{2},n + \frac{1}{2})\backslash \{ n\}$, $\upsilon_{n + 1} \le V'(y)$, by (16), we find

$$\begin{aligned} \omega (\lambda_{2},m) &< \sum _{n = 2}^{\infty} \int_{n - \frac{1}{2}}^{n + \frac{1}{2}} k_{\lambda} \bigl(\ln \alpha U_{m},\ln \beta V(y)\bigr) \frac{\upsilon_{n + 1}\ln^{\lambda_{1}}\alpha U_{m}}{V(y)\ln^{1 - \lambda_{2}}\beta V(y)}\,dy \\ &\le \sum_{n = 2}^{\infty} \int_{n - \frac{1}{2}}^{n + \frac{1}{2}} k_{\lambda} \bigl(\ln \alpha U_{m},\ln \beta V(y)\bigr) \frac{V'(y)\ln^{\lambda_{1}}\alpha U_{m}}{V(y)\ln^{1 - \lambda_{2}}\beta V(y)}\,dy \\ &= \int_{\frac{3}{2}}^{\infty} k_{\lambda} \bigl(\ln \alpha U_{m},\ln \beta V(y)\bigr)\frac{V'(y)\ln^{\lambda_{1}}\alpha U_{m}}{V(y)\ln^{1 - \lambda_{2}}\beta V(y)}\,dy. \end{aligned} $$

Setting $t = \frac{\ln \beta V(y)}{\ln \alpha U_{m}}$ in the above, since $\beta V(\frac{3}{2}) = \beta (1 + \frac{\upsilon_{2}}{2}) \ge 1$ and $\frac{V'(y)}{V(y)}\,dy = (\ln \alpha U_{m})\,dt$, we find

$$\omega (\lambda_{2},m) < \int_{0}^{\infty} k_{\lambda} (1,t)t^{\lambda_{2} - 1}\,dt = K_{\gamma} (\lambda_{1}). $$

Hence, we obtain (18). In the same way, we obtain (19). □

### Note

For example, $\mu_{n} = \upsilon_{n} = \frac{1}{n^{\sigma}}$ ($0 \le \sigma \le 1$) satisfies the conditions of Lemma 2.

### Lemma 3

*With regard to the assumptions of Lemma* 2, (i) *for* $m,n \in \mathbb{N}\backslash \{ 1\}$, *we have*

20 $$\begin{aligned}& K_{\gamma} (\lambda_{1}) \bigl(1 - \theta ( \lambda_{2},m)\bigr) < \omega (\lambda_{2},m), \end{aligned}$$

21 $$\begin{aligned}& K_{\gamma} (\lambda_{1}) \bigl(1 - \vartheta ( \lambda_{1},n)\bigr) < \varpi (\lambda_{1},n), \end{aligned}$$

*where*

22 $$\begin{aligned}& \begin{aligned}[b] \theta (\lambda_{2},m) &= \frac{k_{\lambda} (1,\frac{\ln \beta (1 + \upsilon_{2}\theta (m))}{\ln \alpha U_{m}})}{\lambda_{2}K_{\gamma} (\lambda_{1})}\frac{\ln^{\lambda_{2}}\beta (1 + \upsilon_{2})}{\ln^{\lambda_{2}}\alpha U_{m}}\\ & = O \biggl(\frac{1}{\ln^{\lambda_{2}}\alpha U_{m}}\biggr) \in (0,1)\quad \biggl(\theta (m) \in \biggl( \frac{1 - \beta}{\beta \upsilon_{2}},1\biggr)\biggr), \end{aligned} \end{aligned}$$

23 $$\begin{aligned}& \begin{aligned}[b] \vartheta (\lambda_{1},n) &= \frac{k_{\lambda} (\frac{\ln \alpha (1 + \mu_{2}\vartheta (n))}{\ln \beta V_{n}})}{\lambda_{1}K_{\gamma} (\lambda_{1})}\frac{\ln^{\lambda_{1}}\alpha (1 + \mu_{2})}{\ln^{\lambda_{1}}\beta V_{n}}\\ & = O \biggl(\frac{1}{\ln^{\lambda_{1}}\beta V_{n}}\biggr) \in (0,1)\quad \biggl(\vartheta (n) \in \biggl( \frac{1 - \alpha}{\alpha \mu_{2}},1\biggr)\biggr); \end{aligned} \end{aligned}$$

(ii) *for any* ${c}>0$, *we have*

24 $$\begin{aligned}& \sum_{m = 2}^{\infty} \frac{\mu_{m + 1}}{U_{m}\ln^{1 + c}\alpha U_{m}} = \frac{1}{c} \biggl[ \frac{1}{\ln^{c}\alpha (1 + \mu_{2})} + cO(1) \biggr], \end{aligned}$$

25 $$\begin{aligned}& \sum_{n = 2}^{\infty} \frac{\upsilon_{n + 1}}{V_{n}\ln^{1 + c}\beta V_{n}} = \frac{1}{c} \biggl[ \frac{1}{\ln^{c}\beta (1 + \upsilon_{2})} + c\tilde{O}(1) \biggr]. \end{aligned}$$

### Proof

In view of $\beta \le 1$ and $\beta \ge \frac{1}{1 + \upsilon_{2}/2} > \frac{1}{1 + \upsilon_{2}}$, it follows that $1 \le \frac{1 - \beta}{\beta \upsilon_{2}} + 1 < 2$. Since, by Examples 1, $g(y)$ is strictly decreasing in $[n,n + 1)$, then for $m \in \mathbb{N}\backslash \{ 1\}$, we find

$$\begin{aligned} \omega (\lambda_{2},m) &> \sum _{n = 2}^{\infty} \int_{n}^{n + 1} k_{\lambda} \bigl(\ln \alpha U_{m},\ln \beta V(y)\bigr) \frac{\upsilon_{n + 1}\ln^{\lambda_{1}}\alpha U_{m}}{V(y)\ln^{1 - \lambda_{2}}\beta V(y)}\,dy \\ &= \int_{2}^{\infty} k_{\lambda} \bigl(\ln \alpha U_{m},\ln \beta V(y)\bigr)\frac{V'(y)\ln^{\lambda_{1}}\alpha U_{m}}{V(y)\ln^{1 - \lambda_{2}}\beta V(y)}\,dy \\ &= \int_{\frac{1 - \beta}{\beta \upsilon_{2}} + 1}^{\infty} k_{\lambda} \bigl(\ln \alpha U_{m},\ln \beta V(y)\bigr)\frac{V'(y)\ln^{\lambda_{1}}\alpha U_{m}}{V(y)\ln^{1 - \lambda_{2}}\beta V(y)}\,dy \\ &\quad {}- \int_{\frac{1 - \beta}{\beta \upsilon_{2}} + 1}^{2} k_{\lambda} \bigl(\ln \alpha U_{m},\ln \beta V(y)\bigr)\frac{V'(y)\ln^{\lambda_{1}}\alpha U_{m}}{V(y)\ln^{1 - \lambda_{2}}\beta V(y)}\,dy. \end{aligned} $$

Setting $t = \frac{\ln \beta V(y)}{\ln \alpha U_{m}}$, we have $\ln \beta V(\frac{1 - \beta}{\beta \upsilon_{2}} + 1) = \ln \beta (1 + \frac{1 - \beta}{\beta \upsilon_{2}}\upsilon_{2}) = 0$ and

$$\begin{aligned} \omega (\lambda_{2},m) &> \int_{0}^{\infty} k_{\lambda} (1,t)t^{\lambda_{2} - 1}\,dt - \int_{\frac{1 - \beta}{\beta \upsilon_{2}} + 1}^{2} k_{\lambda} \bigl(\ln \alpha U_{m},\ln \beta V(y)\bigr)\frac{V'(y)\ln^{\lambda_{1}}\alpha U_{m}}{V(y)\ln^{1 - \lambda_{2}}\beta V(y)}\,dy \\ &= K_{\gamma} (\lambda_{1}) \bigl(1 - \theta ( \lambda_{2},m)\bigr), \end{aligned} $$

where

$$\theta (\lambda_{2},m): = \frac{\ln^{\lambda_{1}}\alpha U_{m}}{K_{\gamma} (\lambda_{1})} \int_{\frac{1 - \beta}{\beta \upsilon_{2}} + 1}^{2} k_{\lambda} \bigl(\ln \alpha U_{m},\ln \beta V(y)\bigr)\frac{V'(y)}{V(y)\ln^{1 - \lambda_{2}}\beta V(y)}\,dy \in (0,1). $$

In view of the integral mid-value theorem, for fixed $m \in \mathbb{N}\backslash \{ 1\}$, there exists $\theta (m) \in (\frac{1 - \beta}{\beta \upsilon_{2}},1)$ such that

$$\begin{aligned} \theta (\lambda_{2},m) &= \frac{\ln^{\lambda_{1}}\alpha U_{m}}{K_{\gamma} (\lambda_{1})}k_{\lambda} \bigl(\ln \alpha U_{m},\ln \beta V \bigl(1 + \theta (m)\bigr)\bigr) \int_{\frac{1 - \beta}{\beta \upsilon_{2}} + 1}^{2} \frac{V'(y)}{V(y)\ln^{1 - \lambda_{2}}\beta V(y)}\,dy \\ &= \frac{\ln^{\lambda_{1}}\alpha U_{m}}{\lambda_{2}K_{\gamma} (\lambda_{1})}k_{\lambda} \bigl(\ln \alpha U_{m},\ln \beta V\bigl(1 + \theta (m)\bigr)\bigr)\ln^{\lambda_{2}}\beta (1 + \upsilon_{2}) \\ &= \frac{1}{\lambda_{2}K_{\gamma} (\lambda_{1})}k_{\lambda} \biggl(1,\frac{\ln \beta V(1 + \theta (m))}{\ln \alpha U_{m}}\biggr) \frac{\ln^{\lambda_{2}}\beta (1 + \upsilon_{2})}{\ln^{\lambda_{2}}\alpha U_{m}}. \end{aligned} $$

Hence, we find

$$0 < \theta (\lambda_{2},m) \le \frac{1}{\lambda_{2}K_{\gamma} (\lambda_{1})} \frac{\ln^{\lambda_{2}}\beta (1 + \upsilon_{2})}{(1 + \gamma )\ln^{\lambda_{2}}\alpha U_{m}}, $$

namely, $\theta (\lambda_{2},m) = O(\frac{1}{\ln^{\lambda_{2}}\alpha U_{m}})$. Then we obtain (20) and (22). In the same way, we obtain (21) and (23).

For ${c}>0$, we find

$$\begin{aligned}& \begin{aligned} \sum_{m = 2}^{\infty} \frac{\mu_{m + 1}}{U_{m}\ln^{1 + c}\alpha U_{m}} &\le \sum_{m = 2}^{\infty} \frac{\mu_{m}}{U_{m}\ln^{1 + c}\alpha U_{m}} = \frac{\mu_{2}}{U_{2}\ln^{1 + c}\alpha U_{2}} + \sum_{m = 3}^{\infty} \frac{\mu_{m}}{U_{m}\ln^{1 + c}\alpha U_{m}} \\ &= \frac{\mu_{2}}{U_{2}\ln^{1 + c}\alpha U_{2}} + \sum_{m = 3}^{\infty} \int_{m - 1}^{m} \frac{U'(x)\,dx}{U_{m}\ln^{1 + c}\alpha U_{m}} \\ &< \frac{\mu_{2}}{U_{2}\ln^{1 + c}\alpha U_{2}} + \sum_{m = 3}^{\infty} \int_{m - 1}^{m} \frac{U'(x)\,dx}{U(x)\ln^{1 + c}\alpha U(x)} \\ &= \frac{\mu_{2}}{U_{2}\ln^{1 + c}\alpha U_{2}} + \int_{2}^{\infty} \frac{U'(x)\,dx}{U(x)\ln^{1 + c}\alpha U(x)}\\ & = \frac{\mu_{2}}{U_{2}\ln^{1 + c}\alpha U_{2}} + \frac{1}{c\ln^{c}\alpha (1 + \mu_{2})} \\ &= \frac{1}{c} \biggl[ \frac{1}{\ln^{c}\alpha (1 + \mu_{2})} + c\frac{\mu_{2}}{U_{2}\ln^{1 + c}\alpha U_{2}} \biggr], \end{aligned} \\& \begin{aligned} \sum_{m = 2}^{\infty} \frac{\mu_{m + 1}}{U_{m}\ln^{1 + c}\alpha U_{m}} &= \sum_{m = 2}^{\infty} \int_{m}^{m + 1} \frac{U'(x)\,dx}{U_{m}\ln^{1 + c}\alpha U_{m}} > \sum _{m = 2}^{\infty} \int_{m}^{m + 1} \frac{U'(x)\,dx}{U(x)\ln^{1 + c}\alpha U(x)} \\ &= \int_{2}^{\infty} \frac{U'(x)\,dx}{U(x)\ln^{1 + c}\alpha U(x)} = \frac{1}{c\ln^{c}\alpha (1 + \mu_{2})}. \end{aligned} \end{aligned}$$

Hence, we obtain (20). In the same way, we obtain (21). □

### Lemma 4

*If* $- 1 < \gamma \le 0$, $0 < \lambda_{1},\lambda_{2} < 1$, $\lambda_{1} + \lambda_{2} \le 1$, $K_{\gamma} (\lambda_{1})$ *is determined by* (12), *then for* $0 < \delta < \min \{ \lambda_{1},\lambda_{2}\}$, *we have*

26 $$ K_{\gamma} (\lambda_{1} \pm \delta ) = K_{\gamma} ( \lambda_{1}) + o(1)\quad \bigl(\delta \to 0^{ +} \bigr). $$

### Proof

We find, for $0 < \delta < \min \{ \lambda_{1},\lambda_{2}\}$,

$$\begin{aligned} \bigl\vert K_{\gamma} (\lambda_{1} + \delta ) - K_{\gamma} (\lambda_{1})\bigr\vert &\le \int_{0}^{\infty} \frac{t^{\lambda_{1} - 1}\vert t^{\delta} - 1\vert }{t^{\lambda} + 1 + \gamma \vert t^{\lambda} - 1\vert } \,dt \\ &= \int_{0}^{1} \frac{t^{\lambda_{1} - 1}(1 - t^{\delta} )}{1 + \gamma + (1 - \gamma )t^{\lambda}} \,dt + \int_{1}^{\infty} \frac{t^{\lambda_{1} - 1}(t^{\delta} - 1)}{1 - \gamma + (1 + \gamma )t^{\lambda}} \,dt \\ &\le \frac{1}{1 + \gamma} \biggl[ \int_{0}^{1} t^{\lambda_{1} - 1}\bigl(1 - t^{\delta} \bigr) \,dt + \int_{1}^{\infty} \frac{t^{\lambda_{1} - 1}(t^{\delta} - 1)}{t^{\lambda}} \,dt \biggr] \\ &= \frac{1}{1 + \gamma} \biggl( \frac{1}{\lambda_{1}} - \frac{1}{\lambda_{1} + \delta} + \frac{1}{\lambda_{2} - \delta} - \frac{1}{\lambda_{2}} \biggr) \to 0\quad \bigl(\delta \to 0^{ +} \bigr). \end{aligned} $$

In the same way, we find

$$\begin{aligned} \bigl\vert K_{\gamma} (\lambda_{1} - \delta ) - K_{\gamma} (\lambda_{1})\bigr\vert &\le \frac{1}{1 + \gamma} \biggl[ \int_{0}^{1} t^{\lambda_{1} - 1}\bigl(t^{ - \delta} - 1\bigr) \,dt + \int_{1}^{\infty} \frac{t^{\lambda_{1} - 1}(1 - t^{ - \delta} )}{t^{\lambda}} \,dt \biggr] \\ &= \frac{1}{1 + \gamma} \biggl( \frac{1}{\lambda_{1} - \delta} - \frac{1}{\lambda_{1}} + \frac{1}{\lambda_{2}} - \frac{1}{\lambda_{2} + \delta} \biggr) \to 0\quad \bigl(\delta \to 0^{ +} \bigr), \end{aligned} $$

and then we have (26). □

## Main results

In the following, we also set

27 $$ \begin{gathered} \tilde{\Phi}_{\lambda} (m): = \omega (\lambda_{2},m) \biggl(\frac{U_{m}}{\mu_{m + 1}}\biggr)^{p - 1}( \ln \alpha U_{m})^{p(1 - \lambda_{1}) - 1}\quad \bigl(m \in \mathbb{N}\backslash \{ 1\} \bigr), \\ \tilde{\Psi}_{\lambda} (n): = \varpi (\lambda_{1},n) \biggl( \frac{V_{n}}{\upsilon_{n + 1}}\biggr)^{q - 1}(\ln \beta V_{n})^{q(1 - \lambda_{2}) - 1}\quad \bigl(n \in \mathbb{N}\backslash \{ 1\} \bigr). \end{gathered} $$

### Theorem 1

(i) *For* ${p} >1$, *we have the following equivalent inequalities*:
  28 $$\begin{aligned}& I: = \sum_{n = 2}^{\infty} \sum _{m = 2}^{\infty} k_{\lambda} (\ln \alpha U_{m},\ln \beta V_{n})a_{m}b_{n} \le \Vert a \Vert _{p,\tilde{\Phi}_{\lambda}} \Vert b \Vert _{q,\tilde{\Psi}_{\lambda}}, \end{aligned}$$
  29 $$\begin{aligned}& J: = \Biggl\{ \sum_{n = 2}^{\infty} \frac{\upsilon_{n + 1}\ln^{p\lambda_{2} - 1}\beta V_{n}}{(\varpi (\lambda_{1},n))^{p - 1}V_{n}} \Biggl( \sum_{m = 2}^{\infty} k_{\lambda} (\ln \alpha U_{m},\ln \beta V_{n})a_{m} \Biggr)^{p} \Biggr\} ^{\frac{1}{p}} \le \Vert a \Vert _{p,\tilde{\Phi}_{\lambda}}; \end{aligned}$$
(ii) *for* $0< p<1$ (*or* $p<0$), *we have the equivalent reverse of* (28) *and* (29).

### Proof

(i) By Hölder’s inequality with weight (cf. [20]) and (17), we have

30 $$\begin{aligned}& \Biggl( \sum _{m = 2}^{\infty} k_{\lambda} (\ln \alpha U_{m},\ln \beta V_{n})a_{m} \Biggr)^{p} \\& \quad = \Biggl[ \sum_{m = 2}^{\infty} k_{\lambda} (\ln \alpha U_{m},\ln \beta V_{n}) \biggl( \frac{U_{m}^{1/q}(\ln \alpha U_{m})^{(1 - \lambda_{1})/q}\upsilon_{n + 1}^{1/p}}{(\ln \beta V_{n})^{(1 - \lambda_{2})/p}\mu_{m + 1}^{1/q}}a_{m} \biggr) \\& \qquad {}\times \biggl( \frac{(\ln \beta V_{n})^{(1 - \lambda_{2})/p}\mu_{m + 1}^{1/q}}{U_{m}^{1/q}(\ln \alpha U_{m})^{(1 - \lambda_{1})/q}\upsilon_{n + 1}^{1/p}} \biggr) \Biggr]^{p} \\& \quad \le \sum_{m = 2}^{\infty} k_{\lambda} (\ln \alpha U_{m},\ln \beta V_{n}) \frac{U_{m}^{p - 1}(\ln \alpha U_{m})^{(1 - \lambda_{1})p/q}\upsilon_{n + 1}}{(\ln \beta V_{n})^{1 - \lambda_{2}}\mu_{m + 1}^{p/q}}a_{m}^{p} \\& \qquad {}\times \Biggl[ \sum_{m = 2}^{\infty} k_{\lambda} (\ln \alpha U_{m},\ln \beta V_{n}) \frac{(\ln \beta V_{n})^{(1 - \lambda_{2})(q - 1)}\mu_{m + 1}}{U_{m}(\ln \alpha U_{m})^{1 - \lambda_{1}}\upsilon_{n + 1}^{q - 1}} \Biggr]^{p - 1} \\& \quad = \frac{(\varpi (\lambda_{1},n))^{p - 1}V_{n}}{(\ln \beta V_{n})^{p\lambda_{2} - 1}\upsilon_{n + 1}}\sum_{m = 2}^{\infty} k_{\lambda} (\ln \alpha U_{m},\ln \beta V_{n}) \frac{\upsilon_{n + 1}U_{m}^{p - 1}(\ln \alpha U_{m})^{(1 - \lambda_{1})(p - 1)}}{(\ln \beta V_{n})^{1 - \lambda_{2}}\mu_{m + 1}^{p - 1}}a_{m}^{p}. \end{aligned}$$

Then, by (16), we find

31 $$ \begin{aligned}[b] J &\le \Biggl[ \sum_{n = 2}^{\infty} \sum_{m = 2}^{\infty} k_{\lambda} (\ln \alpha U_{m},\ln \beta V_{n}) \frac{\upsilon_{n + 1}U_{m}^{p - 1}(\ln \alpha U_{m})^{(1 - \lambda_{1})(p - 1)}}{(\ln \beta V_{n})^{1 - \lambda_{2}}\mu_{m + 1}^{p - 1}}a_{m}^{p} \Biggr]^{\frac{1}{p}} \\ &= \Biggl[ \sum_{m = 2}^{\infty} \sum _{n = 2}^{\infty} k_{\lambda} (\ln \alpha U_{m},\ln \beta V_{n}) \frac{\upsilon_{n + 1}U_{m}^{p - 1}(\ln \alpha U_{m})^{(1 - \lambda_{1})(p - 1)}}{(\ln \beta V_{n})^{1 - \lambda_{2}}\mu_{m + 1}^{p - 1}}a_{m}^{p} \Biggr]^{\frac{1}{p}} \\ &= \Biggl[ \sum_{m = 2}^{\infty} \omega ( \lambda_{2},m) \biggl(\frac{U_{m}}{\mu_{m + 1}}\biggr)^{p - 1}(\ln \alpha U_{m})^{p(1 - \lambda_{1}) - 1}a_{m}^{p} \Biggr]^{\frac{1}{p}}, \end{aligned} $$

and then (29) follows.

By Hölder’s inequality (cf. [20]), we have

32 $$ \begin{aligned}[b] I &= \sum_{n = 2}^{\infty} \Biggl[ \frac{(\ln \beta V_{n})^{\lambda_{2} - \frac{1}{p}}\upsilon_{n + 1}^{1/p}}{(\varpi (\lambda_{1},n))^{1/q}V_{n}^{1/p}}\sum_{m = 2}^{\infty} k_{\lambda} (\ln \alpha U_{m},\ln \beta V_{n})a_{m} \Biggr] \biggl[ \frac{(\varpi (\lambda_{1},n))^{1/q}V_{n}^{1/p}}{(\ln \beta V_{n})^{\lambda_{2} - \frac{1}{p}}\upsilon_{n + 1}^{1/p}}b_{n} \biggr] \\ &\le J \Vert b \Vert _{q,\tilde{\Psi}_{\lambda}}.\end{aligned} $$

Then, by (29), we have (28).

On the other hand, assuming that (28) is valid, we set

33 $$ b_{n}: = \frac{\upsilon_{n + 1}\ln^{p\lambda_{2} - 1}\beta V_{n}}{(\varpi (\lambda_{1},n))^{p - 1}V_{n}} \Biggl( \sum _{m = 2}^{\infty} k_{\lambda} (\ln \alpha U_{m},\ln \beta V_{n})a_{m} \Biggr)^{p - 1},\quad n \in \mathbb{N}\backslash \{ 1\}. $$

Then we find $J^{p} = \Vert b \Vert _{q,\tilde{\Psi}_{\lambda}}^{q}$. If $J = 0$, then (29) is trivially valid; if $J = \infty$, then by (31), (29) takes the form of equality. Suppose that $0 < J < \infty$. By (28), it follows that

34 $$\begin{aligned}& \Vert b \Vert _{q,\tilde{\Psi}_{\lambda}}^{q} = J^{p} = I \le \Vert a \Vert _{p,\tilde{\Phi}_{\lambda}} \Vert b \Vert _{q,\tilde{\Psi}_{\lambda}}, \end{aligned}$$

35 $$\begin{aligned}& \Vert b \Vert _{q,\tilde{\Psi}_{\lambda}}^{q - 1} = J \le \Vert a \Vert _{p,\tilde{\Phi}_{\lambda}}, \end{aligned}$$

and then (29) follows, which is equivalent to (28).

(ii) For $0<{p}<1$ (or ${p}<0$), by the reverse Hölder’s inequality with weight (cf. [20]) and (13), we obtain the reverse of (30) (or (30)), then we have the reverse of (31), and then the reverse of (29) follows. By Hölder’s inequality (cf. [20]), we have the reverse of (32), and then by the reverse of (29), the reverse of (28) follows.

On the other hand, assuming that the reverse of (28) is valid, we set $b_{n}$ as (33). Then we find $J^{p} = \Vert b \Vert _{q,\tilde{\Psi}_{\lambda}}^{q}$. If $J = \infty$, then the reverse of (29) is trivially valid; if $J = 0$, then by the reverse of (31), (29) takes the form of equality (=0). Suppose that $0 < J < \infty$. By the reverse of (28), it follows that the reverses of (34) and (35) are valid, and then the reverse of (29) follows, which is equivalent to the reverse of (28). □

### Theorem 2

*If* ${p}>1$, $\{ \mu_{m}\}_{m = 1}^{\infty}$ *and* $\{ \upsilon_{n}\}_{n = 1}^{\infty}$ *are decreasing*, $U_{\infty} = V_{\infty} = \infty$, $\Vert a \Vert _{p,\Phi_{\lambda}} \in \mathbb{R}_{ +}$ *and* $\Vert b \Vert _{q,\Psi_{\lambda}}^{q} \in \mathbb{R}_{ +}$, *then we have the following equivalent inequalities*:

36 $$\begin{aligned}& \sum_{n = 2}^{\infty} \sum _{m = 2}^{\infty} k_{\lambda} (\ln \alpha U_{m},\ln \beta V_{n})a_{m}b_{n} < K_{\gamma} (\lambda_{1}) \Vert a \Vert _{p,\Phi_{\lambda}} \Vert b \Vert _{q,\Psi_{\lambda}}, \end{aligned}$$

37 $$\begin{aligned}& J_{1}: = \Biggl\{ \sum_{n = 2}^{\infty} \frac{\upsilon_{n + 1}}{V_{n}} \ln^{p\lambda_{2} - 1}\beta V_{n} \Biggl( \sum _{m = 2}^{\infty} k_{\lambda} (\ln \alpha U_{m},\ln \beta V_{n})a_{m} \Biggr)^{p} \Biggr\} ^{\frac{1}{p}} < K_{\gamma} (\lambda_{1}) \Vert a \Vert _{p,\Phi_{\lambda}}, \end{aligned}$$

*where the constant factor* $K_{\gamma} (\lambda_{1})$ *is the best possible*.

### Proof

Using (18) and (19) in (28) and (29), we obtain the equivalent inequalities (36) and (37).

For $\varepsilon \in (0,\min \{ p\lambda_{1},p(1 - \lambda_{2})\} )$, we set $\tilde{\lambda}_{1} = \lambda_{1} - \frac{\varepsilon}{p}$ ($\in (0,1)$), $\tilde{\lambda}_{2} = \lambda_{2} + \frac{\varepsilon}{p}$ ($\in (0,1)$), and

38 $$ \begin{gathered}[b] \tilde{a}_{m}: = \frac{\mu_{m + 1}}{U_{m}}\ln^{\tilde{\lambda}_{1} - 1}\alpha U_{m} = \frac{\mu_{m + 1}}{U_{m}} \ln^{\lambda_{1} - \frac{\varepsilon}{p} - 1}\alpha U_{m}, \\ \tilde{b}_{n}: = \frac{\upsilon_{n + 1}}{V_{n}}\ln^{\tilde{\lambda}_{2} - \varepsilon - 1}\beta V_{n} = \frac{\upsilon_{n + 1}}{V_{n}}\ln^{\lambda_{2} - \frac{\varepsilon}{q} - 1}\beta V_{n}. \end{gathered} $$

Then, by (24), (25) and (21), we have

$$\begin{aligned}& \begin{aligned} \Vert \tilde{a} \Vert _{p,\Phi_{\lambda}} \Vert \tilde{b} \Vert _{q,\Psi_{\lambda}} &= \Biggl( \sum_{m = 2}^{\infty} \frac{\mu_{m + 1}}{U_{m}\ln^{1 + \varepsilon} \alpha U_{m}} \Biggr)^{\frac{1}{p}} \Biggl( \sum _{n = 2}^{\infty} \frac{\upsilon_{n + 1}}{V_{n}\ln^{1 + \varepsilon} \beta V_{n}} \Biggr)^{\frac{1}{q}} \\ &= \frac{1}{\varepsilon} \biggl[ \frac{1}{\ln^{\varepsilon} \alpha (1 + \mu_{2})} + \varepsilon O(1) \biggr]^{\frac{1}{p}} \biggl[ \frac{1}{\ln^{\varepsilon} \beta (1 + \upsilon_{2})} + \varepsilon \tilde{O}(1) \biggr]^{\frac{1}{q}}, \end{aligned} \\& \begin{aligned} \tilde{I}&: = \sum_{n = 2}^{\infty} \sum_{m = 2}^{\infty} k_{\lambda} (\ln \alpha U_{m},\ln \beta V_{n})\tilde{a}_{m} \tilde{b}_{n} \\ &= \sum_{n = 2}^{\infty} \Biggl[ \sum _{m = 2}^{\infty} k_{\lambda} (\ln \alpha U_{m},\ln \beta V_{n})\frac{\mu_{m + 1}\ln^{\tilde{\lambda}_{2}}\beta V_{n}}{U_{m}\ln^{1 - \tilde{\lambda}_{1}}\alpha U_{m}} \Biggr] \frac{\upsilon_{n + 1}}{V_{n}}\ln^{ - \varepsilon - 1}\beta V_{n} \\ &= \sum_{n = 2}^{\infty} \frac{\upsilon_{n + 1}\varpi (\lambda_{1},n)}{V_{n}\ln^{\varepsilon + 1}\beta V_{n}} \ge K_{\gamma} (\tilde{\lambda}_{1})\sum _{n = 2}^{\infty} \biggl( 1 - O\biggl(\frac{1}{\ln^{\tilde{\lambda}_{1}}\beta V_{n}} \biggr) \biggr)\frac{\upsilon_{n + 1}}{V_{n}\ln^{\varepsilon + 1}\beta V_{n}} \\ &= K_{\gamma} (\tilde{\lambda}_{1}) \Biggl[ \sum _{n = 2}^{\infty} \frac{\upsilon_{n + 1}}{V_{n}\ln^{\varepsilon + 1}\beta V_{n}} - \sum _{n = 2}^{\infty} O\biggl(\frac{1}{\ln^{\lambda_{1} + \frac{\varepsilon}{q} + 1}\beta V_{n}}\biggr) \frac{\upsilon_{n + 1}}{V_{n}} \Biggr] \\ &= \frac{1}{\varepsilon} K_{\gamma} (\tilde{\lambda}_{1}) \biggl[ \frac{1}{\ln^{\varepsilon} \beta (1 + \upsilon_{2})} + \varepsilon \bigl(\tilde{O}(1) - O(1)\bigr) \biggr]. \end{aligned} \end{aligned}$$

If there exists a positive constant $K \le K_{\gamma} (\lambda_{1})$ such that (36) is valid when replacing $K_{\gamma} (\lambda_{1})$ by *K*, then, in particular, we have $\varepsilon \tilde{I} < \varepsilon K \Vert \tilde{a} \Vert _{p,\Phi_{\lambda}} \Vert \tilde{b} \Vert _{q,\Psi_{\lambda}}$, namely,

$$\begin{aligned}& K_{\gamma} \biggl(\lambda_{1} - \frac{\varepsilon}{p}\biggr) \biggl[ \frac{1}{\ln^{\varepsilon} \beta (1 + \upsilon_{2})} + \varepsilon \bigl( \tilde{O}(1) - O(1)\bigr) \biggr] \\ &\quad < K \biggl[ \frac{1}{\ln^{\varepsilon} \alpha (1 + \mu_{2})} + \varepsilon O(1) \biggr]^{\frac{1}{p}} \biggl[ \frac{1}{\ln^{\varepsilon} \beta (1 + \upsilon_{2})} + \varepsilon \tilde{O}(1) \biggr]^{\frac{1}{q}}. \end{aligned} $$

In view of (26), it follows that $K_{\gamma} (\lambda_{1}) \le K(\varepsilon \to 0^{ +} )$. Hence, $K = K_{\gamma} (\lambda_{1})$ is the best possible constant factor of (36).

Similarly to (32), we still can find the following inequality:

39 $$ I \le J_{1} \Vert b \Vert _{q,\Psi_{\lambda}}. $$

Hence, we can prove that the constant factor $K_{\gamma} (\lambda_{1})$ in (37) is the best possible. Otherwise, we would reach the contradiction by (39) that the constant factor in (36) is not the best possible. □

### Remark 1

(i) For $\alpha = \beta = 1$ in (36) and (37), setting

$$\begin{gathered} \phi_{\lambda} (m): = \biggl(\frac{U_{m}}{\mu_{m + 1}} \biggr)^{p - 1}(\ln U_{m})^{p(1 - \lambda_{1}) - 1}, \\ \psi_{\lambda} (n): = \biggl(\frac{V_{n}}{\upsilon_{n + 1}}\biggr)^{q - 1}(\ln V_{n})^{q(1 - \lambda_{2}) - 1}\quad \bigl(m,n \in \mathbb{N}\backslash \{ 1\} \bigr), \end{gathered} $$

we have the following equivalent Mulholland-type inequalities:

40 $$\begin{aligned}& \sum_{n = 2}^{\infty} \sum _{m = 2}^{\infty} \frac{a_{m}b_{n}}{\ln^{\lambda} U_{m} + \ln^{\lambda} V_{n} + \gamma \vert \ln^{\lambda} U_{m} - \ln^{\lambda} V_{n}\vert } < K_{\gamma} ( \lambda_{1}) \Vert a \Vert _{p,\phi_{\lambda}} \Vert b \Vert _{q,\psi_{\lambda}}, \end{aligned}$$

41 $$\begin{aligned}& \begin{aligned}[b] &\Biggl[ \sum_{n = 2}^{\infty} \frac{\upsilon_{n + 1}}{V_{n}}( \ln V_{n})^{p\lambda_{2} - 1} \Biggl( \sum_{m = 2}^{\infty} \frac{a_{m}}{\ln^{\lambda} U_{m} + \ln^{\lambda} V_{n} + \gamma \vert \ln^{\lambda} U_{m} - \ln^{\lambda} V_{n} \vert } \Biggr)^{p} \Biggr]^{\frac{1}{p}} \\ & \quad < K_{\gamma} (\lambda_{1}) \Vert a \Vert _{p,\phi_{\lambda}}.\end{aligned} \end{aligned}$$

(40) is an extension of (7) and the following inequality (for $\lambda = 1$, $\lambda_{1} = \frac{1}{q}$, $\lambda _{2} = \frac{1}{p}$, $\gamma = 0$):

42 $$ \Biggl[ \sum_{n = 2}^{\infty} \frac{\upsilon_{n + 1}}{V_{n}}( \ln V_{n})^{p\lambda_{2} - 1} \Biggl( \sum_{m = 2}^{\infty} \frac{a_{m}}{\ln U_{m}V_{n}} \Biggr)^{p} \Biggr]^{\frac{1}{p}} < \frac{\pi}{\sin (\frac{\pi}{p})} \Biggl[ \sum_{m = 2}^{\infty} \biggl(\frac{U_{m}}{\mu_{m + 1}}\biggr)^{p - 1}a_{m}^{p} \Biggr]^{\frac{1}{p}}. $$

(ii) For $\lambda = 1$, $\lambda_{1} = \frac{1}{q}$, $\lambda _{2} = \frac{1}{p}$ in (36) and (37), we have the following equivalent inequalities:

43 $$\begin{aligned}& \begin{aligned}[b] &\sum_{n = 2}^{\infty} \sum _{m = 2}^{\infty} \frac{a_{m}b_{n}}{\ln (\alpha \beta U_{m}V_{n}) + \gamma \vert \ln \frac{\alpha U_{m}}{\beta V_{n}} \vert } \\ &\quad < K_{1,\gamma} \biggl(\frac{1}{q}\biggr) \Biggl[ \sum_{m = 2}^{\infty} \biggl(\frac{U_{m}}{\mu_{m + 1}}\biggr)^{p - 1}a_{m}^{p} \Biggr]^{\frac{1}{p}} \Biggl[ \sum_{n = 2}^{\infty} \biggl(\frac{V_{n}}{\upsilon_{n + 1}}\biggr)^{q - 1}b_{n}^{q} \Biggr]^{\frac{1}{q}},\end{aligned} \end{aligned}$$

44 $$\begin{aligned}& \begin{aligned}[b] &\Biggl\{ \sum_{n = 2}^{\infty} \frac{\upsilon_{n + 1}}{V_{n}} \Biggl[ \sum_{m = 2}^{\infty} \frac{a_{m}}{\ln (\alpha \beta U_{m}V_{n}) + \gamma \vert \ln \frac{\alpha U_{m}}{\beta V_{n}}\vert } \Biggr]^{p} \Biggr\} ^{\frac{1}{p}} \\ &\quad < K_{1,\gamma} \biggl( \frac{1}{q}\biggr) \Biggl[ \sum_{m = 2}^{\infty} \biggl(\frac{U_{m}}{\mu_{m + 1}}\biggr)^{p - 1}a_{m}^{p} \Biggr]^{\frac{1}{p}},\end{aligned} \end{aligned}$$

where

45 $$ \begin{aligned}[b] K_{1,\gamma} \biggl(\frac{1}{q} \biggr)&: = \int_{0}^{1} \frac{t^{\frac{ - 1}{p} - 1} + t^{\frac{ - 1}{q} - 1}}{1 + \gamma + (1 - \gamma )t} \,dt = \frac{1}{1 + \gamma} \biggl[ \biggl(\frac{1 + \gamma}{1 - \gamma} \biggr)^{\frac{1}{q}} + \biggl(\frac{1 + \gamma}{1 - \gamma} \biggr)^{\frac{1}{p}} \biggr]\frac{\pi}{\sin (\frac{\pi}{p})} \\ &\quad {}- \frac{1}{1 + \gamma} \sum_{k = 0}^{\infty} ( - 1)^{k} \biggl(\frac{1 + \gamma}{ 1 - \gamma} \biggr)^{k + 1} \biggl( \frac{1}{k + \frac{1}{p}} + \frac{1}{k + \frac{1}{q}} \biggr). \end{aligned} $$

(iii) For $\gamma = 0$, (43) reduces to the following more accurate Hardy-Mulholland-type inequality (7):

46 $$ \sum_{m = 2}^{\infty} \sum _{n = 2}^{\infty} \frac{a_{m}b{}_{n}}{\ln (\alpha \beta U_{m}V_{n})} < \frac{\pi}{\sin (\frac{\pi}{p})} \Biggl[ \sum_{m = 2}^{\infty} \biggl( \frac{U_{m}}{\mu_{m + 1}}\biggr)^{p - 1}a_{m}^{p} \Biggr]^{\frac{1}{p}} \Biggl[ \sum_{n = 2}^{\infty} \biggl(\frac{V_{n}}{\upsilon_{n + 1}}\biggr)^{q - 1}b_{n}^{q} \Biggr]^{\frac{1}{q}}. $$

In particular, for $\mu_{i} = \upsilon_{j} = 1$ ($i,j \in \mathbb{N}$), (46) reduces to the following more accurate Mulholland’s inequality ($\frac{2}{3} \le \alpha,\beta \le 1$):

47 $$ \sum_{m = 2}^{\infty} \sum _{n = 2}^{\infty} \frac{a_{m}b{}_{n}}{\ln (\alpha \beta mn)} < \frac{\pi}{\sin (\frac{\pi}{p})} \Biggl( \sum_{m = 2}^{\infty} m^{p - 1}a_{m}^{p} \Biggr)^{\frac{1}{p}} \Biggl( \sum_{n = 2}^{\infty} n^{q - 1}b_{n}^{q} \Biggr)^{\frac{1}{q}}. $$

For $p > 1$, $\Psi_{\lambda}^{1 - p}(n) = \frac{\upsilon_{n + 1}}{V_{n}}(\ln \beta V_{n})^{p\lambda_{2} - 1}$, we define the following normed spaces:

$$\begin{gathered} l_{p,\Phi_{\lambda}}: = \bigl\{ a = \{ a_{m} \}_{m = 2}^{\infty}; \Vert a \Vert _{p,\Phi_{\lambda}} < \infty \bigr\} , \\ l_{q,\Psi_{\lambda}}: = \bigl\{ b = \{ b_{n}\}_{n = 2}^{\infty}; \Vert b \Vert _{q,\Psi_{\lambda}} < \infty \bigr\} , \\ l_{p,\Psi_{\lambda}^{1 - p}}: = \bigl\{ c = \{ c_{n}\}_{n = 2}^{\infty}; \Vert c \Vert _{p,\Psi_{\lambda}^{1 - p}} < \infty \bigr\} . \end{gathered} $$

Assuming that $a = \{ a_{m}\}_{m = 2}^{\infty} \in l_{p,\Phi_{\lambda}}$, setting

$$c = \{ c_{n}\}_{n = 2}^{\infty},\quad c_{n}: = \sum _{m = 2}^{\infty} k_{\lambda} (\ln \alpha U_{m},\ln \beta V_{n})a_{m},n \in \mathbb{N} \backslash \{ 1\}, $$

we can rewrite (37) as follows:

$$\Vert c \Vert _{p,\Psi_{\lambda}^{1 - p}} < K_{\gamma} (\lambda_{1}) \Vert a \Vert _{p,\Phi_{\lambda}} < \infty, $$

namely, $c \in l_{p,\Psi_{\lambda}^{1 - p}}$.

### Definition 2

Define a Hardy-Mulholland-type operator $T:l_{p,\Phi_{\lambda}} \to l_{p,\Psi_{\lambda}^{1 - p}}$ as follows: For any $a = \{ a_{m}\}_{m = 2}^{\infty} \in l_{p,\Phi_{\lambda}}$, there exists a unique representation $Ta = c \in l_{p,\Psi_{\lambda}^{1 - p}}$. Define the formal inner product of *Ta* and $b = \{ b_{n}\}_{n = 2}^{\infty} \in l_{q,\Psi_{\lambda}}$ as follows:

48 $$ (Ta,b): = \sum_{n = 2}^{\infty} \Biggl( \sum _{m = 2}^{\infty} k_{\lambda} (\ln \alpha U_{m},\ln \beta V_{n})a_{m} \Biggr) b_{n}. $$

Then we can rewrite (36) and (37) as follows:

49 $$\begin{aligned}& (Ta,b) < K_{\gamma} (\lambda_{1}) \Vert a \Vert _{p,\Phi_{\lambda}} \Vert b \Vert _{q,\Psi_{\lambda}}, \end{aligned}$$

50 $$\begin{aligned}& \Vert Ta \Vert _{p,\Psi_{\lambda}^{1 - p}} < K_{\gamma} (\lambda_{1}) \Vert a \Vert _{p,\Phi_{\lambda}}. \end{aligned}$$

Define the norm of operator *T* as follows:

$$\Vert T \Vert : = \sup_{a( \ne \theta ) \in l_{p,\Phi_{\lambda}}} \frac{ \Vert Ta \Vert _{p,\Psi_{\lambda}^{1 - p}}}{ \Vert a \Vert _{p,\Phi_{\lambda}}}. $$

Then, by (50), we find $\Vert T \Vert \le K_{\gamma} (\lambda_{1})$. Since the constant factor in (50) is the best possible, we have

51 $$ \Vert T \Vert = K_{\gamma} (\lambda_{1}) = \int_{0}^{1} \frac{t^{\lambda_{1} - 1} + t^{\lambda_{2} - 1}}{1 + \gamma + (1 - \gamma )t^{\lambda}} \,dt. $$

## Some reverses

In the following, we also set

52 $$ \begin{gathered} \tilde{\Omega}_{\lambda} (m): = \bigl(1 - \theta (\lambda_{2},m)\bigr) \biggl(\frac{U_{m}}{\mu_{m + 1}} \biggr)^{p - 1}(\ln \alpha U_{m})^{p(1 - \lambda_{1}) - 1}, \\ \tilde{\Upsilon}_{\lambda} (n): = \bigl(1 - \vartheta ( \lambda_{1},n)\bigr) \biggl(\frac{V_{n}}{\upsilon_{n + 1}}\biggr)^{q - 1}( \ln \beta V_{n})^{q(1 - \lambda_{2}) - 1}\quad \bigl(m,n \in \mathbb{N}\backslash \{ 1 \} \bigr). \end{gathered} $$

For $0 < p < 1$ or $p < 0$, we still use the formal symbols $\Vert a \Vert _{p,\Phi_{\lambda}}$, $\Vert b \Vert _{q,\Psi_{\lambda}}$, $\Vert a \Vert _{p,\tilde{\Omega}_{\lambda}}$ and $\Vert b \Vert _{q,\tilde{\Upsilon}_{\lambda}}$ et al.

### Theorem 3

*If* $0 < p < 1$, $\{ \mu_{m}\}_{m = 1}^{\infty}$ *and* $\{ \upsilon_{n}\}_{n = 1}^{\infty}$ *are decreasing*, $U_{\infty} = V_{\infty} = \infty$, $\Vert a \Vert _{p,\Phi_{\lambda}} \in \mathbb{R}_{ +}$ *and* $\Vert b \Vert _{q,\Psi_{\lambda}}^{q} \in \mathbb{R}_{ +}$, *then we have the following equivalent inequalities with the best possible constant factor* $K_{\gamma} (\lambda_{1})$:

53 $$\begin{aligned}& \sum_{n = 2}^{\infty} \sum _{m = 2}^{\infty} k_{\lambda} (\ln \alpha U_{m},\ln \beta V_{n})a_{m}b_{n} > K_{\gamma} (\lambda_{1}) \Vert a \Vert _{p,\tilde{\Omega}_{\lambda}} \Vert b \Vert _{q,\Psi_{\lambda}}, \end{aligned}$$

54 $$\begin{aligned}& \Biggl\{ \sum_{n = 2}^{\infty} \frac{\upsilon_{n + 1}}{V_{n}} \ln^{p\lambda_{2} - 1}\beta V_{n} \Biggl( \sum _{m = 2}^{\infty} k_{\lambda} (\ln \alpha U_{m},\ln \beta V_{n})a_{m} \Biggr)^{p} \Biggr\} ^{\frac{1}{p}} > K_{\gamma} (\lambda_{1}) \Vert a \Vert _{p,\tilde{\Omega}_{\lambda}}. \end{aligned}$$

### Proof

Using (20) and (19) in the reverses of (28) and (29), since

$$\begin{gathered} \bigl(\omega (\lambda_{2},m) \bigr)^{\frac{1}{p}} > \bigl(K_{\gamma} (\lambda_{1}) \bigr)^{\frac{1}{p}}\bigl(1 - \theta (\lambda_{2},m) \bigr)^{\frac{1}{p}}\quad (0 < p < 1), \\ \bigl(\varpi (\lambda_{1},n)\bigr)^{\frac{1}{q}} > \bigl(K_{\gamma} (\lambda_{1})\bigr)^{\frac{1}{q}}\quad (q < 0), \end{gathered} $$

and

$$\frac{1}{(K_{\gamma} (\lambda_{1}))^{p - 1}} > \frac{1}{(\varpi (\lambda_{1},n))^{p - 1}}\quad (0 < p < 1), $$

we obtain equivalent inequalities (53) and (54).

For $\varepsilon \in (0,\min \{ p\lambda_{1},p(1 - \lambda_{2})\})$, we set $\tilde{\lambda}_{1}$, $\tilde{\lambda}_{2}$, $\tilde{a}_{m}$ and $\tilde{b}_{n}$ as (38). Then, by (24), (25) and (19), we find

$$\begin{aligned}& \Vert \tilde{a} \Vert _{p,\tilde{\Omega}_{\lambda}} \Vert \tilde{b} \Vert _{q,\Psi_{\lambda}} \\& \quad = \Biggl( \sum_{m = 2}^{\infty} \frac{(1 - \theta (\lambda_{2},m))\mu_{m + 1}}{U_{m}\ln^{1 + \varepsilon} \alpha U_{m}} \Biggr)^{\frac{1}{p}} \Biggl( \sum _{n = 2}^{\infty} \frac{\upsilon_{n + 1}}{V_{n}\ln^{1 + \varepsilon} \beta V_{n}} \Biggr)^{\frac{1}{q}} \\& \quad = \Biggl( \sum_{m = 2}^{\infty} \frac{\mu_{m + 1}}{U_{m}\ln^{1 + \varepsilon} \alpha U_{m}} - \sum_{m = 2}^{\infty} O \biggl(\frac{\mu_{m + 1}}{U_{m}\ln^{1 + (\lambda_{2} + \varepsilon )}\alpha U_{m}}\biggr) \Biggr)^{\frac{1}{p}} \Biggl( \sum _{n = 2}^{\infty} \frac{\upsilon_{n + 1}}{V_{n}\ln^{1 + \varepsilon} \beta V_{n}} \Biggr)^{\frac{1}{q}} \\& \quad = \frac{1}{\varepsilon} \biggl[ \frac{1}{\ln^{\varepsilon} \alpha (1 + \mu_{2})} + \varepsilon (O(1) - O_{1}(1) \biggr]^{\frac{1}{p}} \biggl[ \frac{1}{\ln^{\varepsilon} \beta (1 + \upsilon_{2})} + \varepsilon \tilde{O}(1) \biggr]^{\frac{1}{q}}, \\& \begin{aligned} \tilde{I}&: = \sum_{n = 2}^{\infty} \sum_{m = 2}^{\infty} k_{\lambda} (\ln \alpha U_{m},\ln \beta V_{n})\tilde{a}_{m} \tilde{b}_{n} \\ &= \sum_{n = 2}^{\infty} \Biggl[ \sum _{m = 2}^{\infty} k_{\lambda} (\ln \alpha U_{m},\ln \beta V_{n})\frac{\mu_{m + 1}\ln^{\tilde{\lambda}_{2}}\beta V_{n}}{U_{m}\ln^{1 - \tilde{\lambda}_{1}}\alpha U_{m}} \Biggr] \frac{\upsilon_{n + 1}}{V_{n}}\ln^{ - \varepsilon - 1}\beta V_{n} \\ &= \sum_{n = 2}^{\infty} \frac{\upsilon_{n + 1}\varpi (\tilde{\lambda}_{1},n)}{V_{n}\ln^{\varepsilon + 1}\beta V_{n}} \le K_{\gamma} (\tilde{\lambda}_{1})\sum _{n = 2}^{\infty} \frac{\upsilon_{n + 1}}{V_{n}\ln^{\varepsilon + 1}\beta V_{n}} \\ &= \frac{1}{\varepsilon} K_{\gamma} (\tilde{\lambda}_{1}) \biggl[ \frac{1}{\ln^{\varepsilon} \beta (1 + \upsilon_{2})} + \varepsilon \tilde{O}(1) \biggr]. \end{aligned} \end{aligned}$$

If there exists a positive constant $K \ge K_{\gamma} (\lambda_{1})$ such that (53) is valid when replacing $K_{\gamma} (\lambda_{1})$ by *K*, then, in particular, we have $\varepsilon \tilde{I} > \varepsilon K \Vert \tilde{a} \Vert _{p,\Phi_{\lambda}} \Vert \tilde{b} \Vert _{q,\Psi_{\lambda}}$, namely,

$$\begin{gathered} K_{\gamma} \biggl(\lambda_{1} - \frac{\varepsilon}{p}\biggr) \biggl[ \frac{1}{\ln^{\varepsilon} \beta (1 + \upsilon_{2})} + \varepsilon \tilde{O}(1) \biggr] \\ \quad > K \biggl[ \frac{1}{\ln^{\varepsilon} \alpha (1 + \mu_{2})} + \varepsilon \bigl(O(1) - O_{1}(1) \bigr) \biggr]^{\frac{1}{p}} \biggl[ \frac{1}{\ln^{\varepsilon} \beta (1 + \upsilon_{2})} + \varepsilon \tilde{O}(1) \biggr]^{\frac{1}{q}}. \end{gathered} $$

It follows that $K_{\gamma} (\lambda_{1}) \ge K$ ($\varepsilon \to 0^{ +} $). Hence, $K = K_{\gamma} (\lambda_{1})$ is the best possible constant factor of (53).

The constant factor $K_{\gamma} (\lambda_{1})$ in (54) is still the best possible. Otherwise, we would reach the contradiction by the reverse of (39) that the constant factor in (53) is not the best possible. □

### Remark 2

For $\alpha = \beta = 1$, set

$$\begin{gathered} \tilde{\theta} (\lambda_{2},m) = \frac{k_{\lambda} (1,\frac{\ln (1 + \upsilon_{2}\theta (m))}{\ln U_{m}})}{\lambda_{2}K_{\gamma} (\lambda_{1})}\frac{\ln^{\lambda_{2}}(1 + \upsilon_{2})}{ln^{\lambda_{2}}U_{m}} = O\biggl(\frac{1}{ln^{\lambda_{2}}U_{m}}\biggr) \in (0,1)\quad \bigl(\theta (m) \in (0,1)\bigr), \\ \tilde{\phi}_{\lambda} (m): = \bigl(1 - \tilde{\theta} ( \lambda_{2},m)\bigr) \biggl(\frac{U_{m}}{\mu_{m + 1}}\biggr)^{p - 1}( \ln U_{m})^{p(1 - \lambda_{1}) - 1}. \end{gathered} $$

It is evident that (53) and (54) are extensions of the following equivalent inequalities:

55 $$\begin{aligned}& \sum_{n = 2}^{\infty} \sum _{m = 2}^{\infty} k_{\lambda} (\ln U_{m},\ln V_{n})a_{m}b_{n} > K_{\gamma} ( \lambda_{1}) \Vert a \Vert _{p,\tilde{\phi}_{\lambda}} \Vert b \Vert _{q,\psi_{\lambda}}, \end{aligned}$$

56 $$\begin{aligned}& \Biggl\{ \sum_{n = 2}^{\infty} \frac{\upsilon_{n + 1}}{V_{n}} \ln^{p\lambda_{2} - 1}V_{n} \Biggl( \sum_{m = 2}^{\infty} k_{\lambda} (\ln U_{m},\ln V_{n})a_{m} \Biggr)^{p} \Biggr\} ^{\frac{1}{p}} > K_{\gamma} ( \lambda_{1}) \Vert a \Vert _{p,\tilde{\phi}_{\lambda}}, \end{aligned}$$

where the constant factor $K_{\gamma} (\lambda_{1})$ is the best possible.

### Theorem 4

*If* $p < 0$, $\{ \mu_{m}\}_{m = 1}^{\infty}$ *and* $\{ \upsilon_{n}\}_{n = 1}^{\infty}$ *are decreasing*, $U_{\infty} = V_{\infty} = \infty$, $\Vert a \Vert _{p,\Phi_{\lambda}} \in \mathbb{R}_{ +}$ *and* $\Vert b \Vert _{q,\Psi_{\lambda}}^{q} \in \mathbb{R}_{ +}$, *then we have the following equivalent inequalities with the best possible constant factor* $K_{\gamma} (\lambda_{1})$:

57 $$\begin{aligned}& \sum_{n = 2}^{\infty} \sum _{m = 2}^{\infty} k_{\lambda} (\ln \alpha U_{m},\ln \beta V_{n})a_{m}b_{n} > K_{\gamma} (\lambda_{1}) \Vert a \Vert _{p,\Phi_{\lambda}} \Vert b \Vert _{q,\tilde{\Upsilon}_{\lambda}}, \end{aligned}$$

58 $$\begin{aligned}& J_{2}: = \Biggl\{ \sum_{n = 2}^{\infty} \frac{\upsilon_{n + 1}\ln^{p\lambda_{2} - 1}\beta V_{n}}{(1 - \vartheta (\lambda_{1},n))^{p - 1}V_{n}} \Biggl( \sum_{m = 2}^{\infty} k_{\lambda} (\ln \alpha U_{m},\ln \beta V_{n})a_{m} \Biggr)^{p} \Biggr\} ^{\frac{1}{p}} > K_{\gamma} ( \lambda_{1}) \Vert a \Vert _{p,\Phi_{\lambda}}. \end{aligned}$$

### Proof

Using (18) and (21) in the reverses of (28) and (29), since

$$\begin{gathered} \bigl(\omega (\lambda_{2},m) \bigr)^{\frac{1}{p}} > \bigl(K_{\gamma} (\lambda_{1}) \bigr)^{\frac{1}{p}}\quad (p < 0), \\ \bigl(\varpi (\lambda_{1},n)\bigr)^{\frac{1}{q}} > \bigl(K_{\gamma} (\lambda_{1})\bigr)^{\frac{1}{q}}\bigl(1 - \vartheta (\lambda_{1},n)\bigr)^{\frac{1}{q}}\quad (0 < q < 1), \end{gathered} $$

and

$$\biggl[\frac{1}{(K_{\gamma} (\lambda_{1}))^{p - 1}(1 - \vartheta (\lambda_{1},n))^{p - 1}}\biggr]^{\frac{1}{p}} > \biggl[\frac{1}{(\varpi (\lambda_{1},n))^{p - 1}} \biggr]^{\frac{1}{p}}\quad (p < 0), $$

we obtain equivalent inequalities (57) and (58).

For $\varepsilon \in (0,\min \{ q\lambda_{2},q(1 - \lambda_{1})\})$, we set $\tilde{\lambda}_{1} = \lambda_{1} + \frac{\varepsilon}{q}$ ($\in (0,1)$), $\tilde{\lambda}_{2} = \lambda_{2} - \frac{\varepsilon}{q}$ ($\in (0,1)$), and

$$\begin{gathered} \tilde{a}_{m}: = \frac{\mu_{m + 1}}{U_{m}} \ln^{\tilde{\lambda}_{1} - \varepsilon - 1}\alpha U_{m} = \frac{\mu_{m + 1}}{U_{m}}\ln^{\lambda_{1} - \frac{\varepsilon}{p} - 1} \alpha U_{m}, \\ \tilde{b}_{n}: = \frac{\upsilon_{n + 1}}{V_{n}}\ln^{\tilde{\lambda}_{2} - \varepsilon - 1}\beta V_{n} = \frac{\upsilon_{n + 1}}{V_{n}}\ln^{\lambda_{2} - \frac{\varepsilon}{q} - 1}\beta V_{n}. \end{gathered} $$

Then, by (24), (25) and (18), we have

$$\begin{aligned}& \begin{gathered} \Vert \tilde{a} \Vert _{p,\Phi_{\lambda}} \Vert \tilde{b} \Vert _{q,\tilde{\Upsilon}_{\lambda}} \\ \quad = \Biggl( \sum_{m = 2}^{\infty} \frac{\mu_{m + 1}}{U_{m}\ln^{1 + \varepsilon} \alpha U_{m}} \Biggr)^{\frac{1}{p}} \Biggl( \sum _{n = 2}^{\infty} \frac{(1 - \vartheta (\lambda_{1},n))\upsilon_{n + 1}}{V_{n}\ln^{1 + \varepsilon} \beta V_{n}} \Biggr)^{\frac{1}{q}} \\ \quad = \Biggl( \sum_{m = 2}^{\infty} \frac{\mu_{m + 1}}{U_{m}\ln^{1 + \varepsilon} \alpha U_{m}} \Biggr)^{\frac{1}{p}} \Biggl( \sum _{n = 2}^{\infty} \frac{\upsilon_{n + 1}}{V_{n}\ln^{1 + \varepsilon} \beta V_{n}} - \sum _{n = 2}^{\infty} O\biggl(\frac{\upsilon_{n + 1}}{V_{n}\ln^{1 + \varepsilon} \beta V_{n}}\biggr) \Biggr)^{\frac{1}{q}} \\ \quad = \frac{1}{\varepsilon} \biggl[ \frac{1}{\ln^{\varepsilon} \alpha (1 + \mu_{2})} + \varepsilon O(1) \biggr]^{\frac{1}{p}} \biggl[ \frac{1}{\ln^{\varepsilon} \beta (1 + \upsilon_{2})} + \varepsilon \bigl(\tilde{O}(1) - O_{1}(1)\bigr) \biggr]^{\frac{1}{q}}, \end{gathered} \\& \begin{aligned} \tilde{I} &= \sum_{n = 2}^{\infty} \sum_{m = 2}^{\infty} k_{\lambda} (\ln \alpha U_{m},\ln \beta V_{n})\tilde{a}_{m} \tilde{b}_{n} \\ &= \sum_{m = 2}^{\infty} \Biggl[ \sum _{n = 2}^{\infty} k_{\lambda} (\ln \alpha U_{m},\ln \beta V_{n})\frac{\upsilon_{n + 1}\ln^{\tilde{\lambda}_{1}}\alpha U_{m}}{V_{n}\ln^{1 - \tilde{\lambda}_{2}}\beta V_{n}} \Biggr] \frac{\mu_{m + 1}}{U_{m}\ln^{1 + \varepsilon} \alpha U_{m}} \\ &= \sum_{m = 2}^{\infty} \frac{\mu_{m + 1}\omega (\tilde{\lambda}_{2},m)}{U_{m}\ln^{\varepsilon + 1}\alpha U_{m}} \le K_{\gamma} (\tilde{\lambda}_{1})\sum _{m = 2}^{\infty} \frac{\mu_{m + 1}}{U_{m}\ln^{\varepsilon + 1}\alpha U_{m}} \\ &= \frac{1}{\varepsilon} K_{\gamma} (\tilde{\lambda}_{1}) \biggl[ \frac{1}{\ln^{\varepsilon} \beta (1 + \upsilon_{2})} + \varepsilon O(1) \biggr]. \end{aligned} \end{aligned}$$

If there exists a positive constant $K \ge K_{\gamma} (\lambda_{1})$ such that (57) is valid when replacing $K_{\gamma} (\lambda_{1})$ by *K*, then, in particular, we have $\varepsilon \tilde{I} > \varepsilon K \Vert \tilde{a} \Vert _{p,\Phi_{\lambda}} \Vert \tilde{b} \Vert _{q,\tilde{\Upsilon}_{\lambda}}$, namely,

$$\begin{gathered} K_{\gamma} \biggl(\lambda_{1} + \frac{\varepsilon}{q}\biggr) \biggl[ \frac{1}{\ln^{\varepsilon} \alpha (1 + \mu_{2})} + \varepsilon O(1) \biggr] \\ \quad > K \biggl[ \frac{1}{\ln^{\varepsilon} \alpha (1 + \mu_{2})} + \varepsilon O(1) \biggr]^{\frac{1}{p}} \biggl[ \frac{1}{\ln^{\varepsilon} \beta (1 + \upsilon_{2})} + \varepsilon \bigl(\tilde{O}(1) - O_{1}(1)\bigr) \biggr]^{\frac{1}{q}}. \end{gathered} $$

It follows that $K_{\gamma} (\lambda_{1}) \ge K$ ($\varepsilon \to 0^{ +} $). Hence, $K = K_{\gamma} (\lambda_{1})$ is the best possible constant factor of (57).

Similarly to the reverse of (32), we still can find that

59 $$ I \ge J_{2} \Vert b \Vert _{q,\tilde{\Upsilon}_{\lambda}}. $$

Hence, the constant factor $K_{\gamma} (\lambda_{1})$ in (58) is still the best possible. Otherwise, we would reach the contradiction by (59) that the constant factor in (57) is not the best possible. □

### Remark 3

For $\alpha = \beta = 1$, set

$$\begin{gathered} \tilde{\vartheta} (\lambda_{1},n) = \frac{k_{\lambda} (\frac{\ln (1 + \mu_{2}\vartheta (n))}{\ln U_{m}},1)}{\lambda_{1}K_{\gamma} (\lambda_{1})}\frac{\ln^{\lambda_{1}}(1 + \mu_{2})}{ln^{\lambda_{1}}\beta V_{n}} = O\biggl(\frac{1}{ln^{\lambda_{2}}U_{m}}\biggr) \in (0,1)\quad \bigl(\vartheta (n) \in (0,1)\bigr), \\ \tilde{\psi}_{\lambda} (n): = \bigl(1 - \tilde{\vartheta} ( \lambda_{1},n)\bigr) \biggl(\frac{V_{n}}{\upsilon_{n + 1}}\biggr)^{q - 1}( \ln V_{n})^{q(1 - \lambda_{2}) - 1}. \end{gathered} $$

It is evident that (57) and (58) are extensions of the following equivalent inequalities:

60 $$\begin{aligned}& \sum_{n = 2}^{\infty} \sum _{m = 2}^{\infty} k_{\lambda} (\ln U_{m},\ln V_{n})a_{m}b_{n} > K_{\gamma} ( \lambda_{1}) \Vert a \Vert _{p,\phi_{\lambda}} \Vert b \Vert _{q,\tilde{\psi}_{\lambda}}, \end{aligned}$$

61 $$\begin{aligned}& \Biggl\{ \sum_{n = 2}^{\infty} \frac{\upsilon_{n + 1}\ln^{p\lambda_{2} - 1}V_{n}}{V_{n}(1 - \tilde{\vartheta} (\lambda_{1},n))} \Biggl( \sum_{m = 2}^{\infty} k_{\lambda} (\ln U_{m},\ln V_{n})a_{m} \Biggr)^{p} \Biggr\} ^{\frac{1}{p}} > K_{\gamma} ( \lambda_{1}) \Vert a \Vert _{p,\phi_{\lambda}}, \end{aligned}$$

where the constant factor $K_{\gamma} (\lambda_{1})$ is the best possible.

## Conclusions

In this paper, by using the way of weight coefficients, the technique of real analysis, and Hermite-Hadamard’s inequality, a more accurate Hardy-Mulholland-type inequality with multi-parameters and a best possible constant factor is given by Theorems 1, 2, and the equivalent forms are considered. The equivalent reverses with the best possible constant factor are obtained by Theorems 3, 4. Moreover, the operator expressions and some particular cases are considered. The method of weight coefficients is very important, which helps us to prove the main inequalities with the best possible constant factor. The lemmas and theorems provide an extensive account of this type of inequalities.
